# Improved potency of F10 relative to 5-fluorouracil in colorectal cancer cells with p53 mutations

**DOI:** 10.20517/cdr.2018.01

**Published:** 2018-03-19

**Authors:** Anthony Dominijanni, William H. Gmeiner

**Affiliations:** Department of Cancer Biology, Wake Forest School of Medicine, Winston-Salem, NC 27157, USA.

**Keywords:** Fluoropyrimidine, colorectal cancer, p53, drug resistance

## Abstract

**Aim:**

Resistance to fluoropyrimidine drugs (FPs) is a major cause of mortality in colorectal cancer (CRC). We assessed the potency advantage of the polymeric FP F10 relative to 5-fluorouracil (5FU) in four human CRC cell lines that differ only in *TP53* mutational status to determine how p53 mutations affect drug response and whether F10 is likely to improve outcomes.

**Methods:**

HCT-116 human CRC cells (p53^+/+^) and three isogenic variants (p53^−/−^, R248W/+, R248W/−) were assessed for drug response. Resistance factors were derived from cell viability data and used to establish the relative potency advantage for F10. Rescue studies with exogenous uridine/thymidine determined if cytotoxicity resulted from DNA-directed processes.

**Results:**

Significant resistance to 5-FU resulted from p53-loss or from gain-of-function (GOF) mutation (R248W) and was greatest when GOF mutation was coupled with loss of wild-type p53. F10 is much more potent than 5-FU (137–314-fold depending on *TP53* mutational status). F10 and 5-FU induce apoptosis by DNA- and RNA-directed mechanisms, respectively, and only F10 shows a modest enhancement in cytotoxicity upon co-treatment with leucovorin.

**Conclusion:**

*TP53* mutational status affects inherent sensitivity to FPs, with p53 GOF mutations most deleterious. F10 is much more effective than 5-FU regardless of *TP53* mutations and has potential to be effective to CRC that is resistant to 5-FU due, in part, to *TP53* mutations.^6^,^7^

## INTRODUCTION

Colorectal cancer (CRC) is the 3rd leading cause of cancer-related deaths among both men and women. The extreme genetic heterogeneity of CRC, both intra- and intertumoral, makes implementing targeted therapies a challenge^[1]^. Ras mutations and DNA mismatch repair (MMR) status are the predominant biomarkers used to direct clinical decisions^[2]^. Standard-of-care for CRC patients with *KRAS* mutations (~50% of CRC) is 5-fluorouracil (5-FU)-based chemotherapy, except for the small percent of patients with microsatellite instability (MSI - a result of MMR deficiency) that do not respond to 5-FU-based regimens^[2]^. Despite considerable progress made using fluoropyrimidine drugs (FPs), mainly 5-FU, that constitute the backbone of combination chemotherapy regimens for treating CRC (e.g. FOLFOX; FOLFIRI) and provide a survival benefit^[3]^ for patients with stage II, III, and IV CRC, there remains a sizable fraction of patients with recurrent disease (~30%^[4]^), and a continued need to develop improved therapies.

Factors responsible for disease recurrence in CRC patients treated with 5-FU remain incompletely characterized, however growing evidence implicates *TP53* mutations, which occur in up to half of CRC cases, as an important risk factor^[5-8]^. At a molecular level, cellular response to 5-FU is *TP53*-dependent^[9]^. Non-malignant gastrointestinal (GI-) tract cells expressing wild-type (*wtTP53*) undergo apoptosis in response to 5-FU^[10]^, contributing to drug toxicity. *TP53*-dependent apoptosis also occurs in CRC tumor cells^[11,12]^ and 5-FU’s cytotoxic effects may be partly rescued by uridine (Urd) in some CRC cell lines consistent with an RNA-directed process^[13,14]^. While *TP53* status is important for response of GI-tract and CRC cells to 5-FU, investigations to test whether *TP53* status predicts therapy response in CRC have yielded conflicting results^[8,15]^, possibly due to different methods being used to assess *TP53* mutational status^[16]^. One recent clinical study conducted using a novel p53-specific sequencing protocol concluded *TP53* mutations were associated with 5-FU refractory disease in stage III CRC patients^[8]^. One possible contributing factor for how *TP53* status might affect disease recurrence is through a sub-set of *TP53* missense mutations that confer gain-of-function (GOF^[17]^) and that activate pro-metastatic signaling pathways^[18,19]^, yet may inhibit druginduced, p21-dependent apoptosis^[20,21]^. While further clinical investigation is required to assess whether *TP53* mutational status should be used to direct therapy decisions in CRC based on 5-FU response, it is important to establish that candidates for replacing 5-FU demonstrate strong activity towards CRC with p53 mutations since this constitutes 40%−50% of CRC cases.

**Figure 1:**
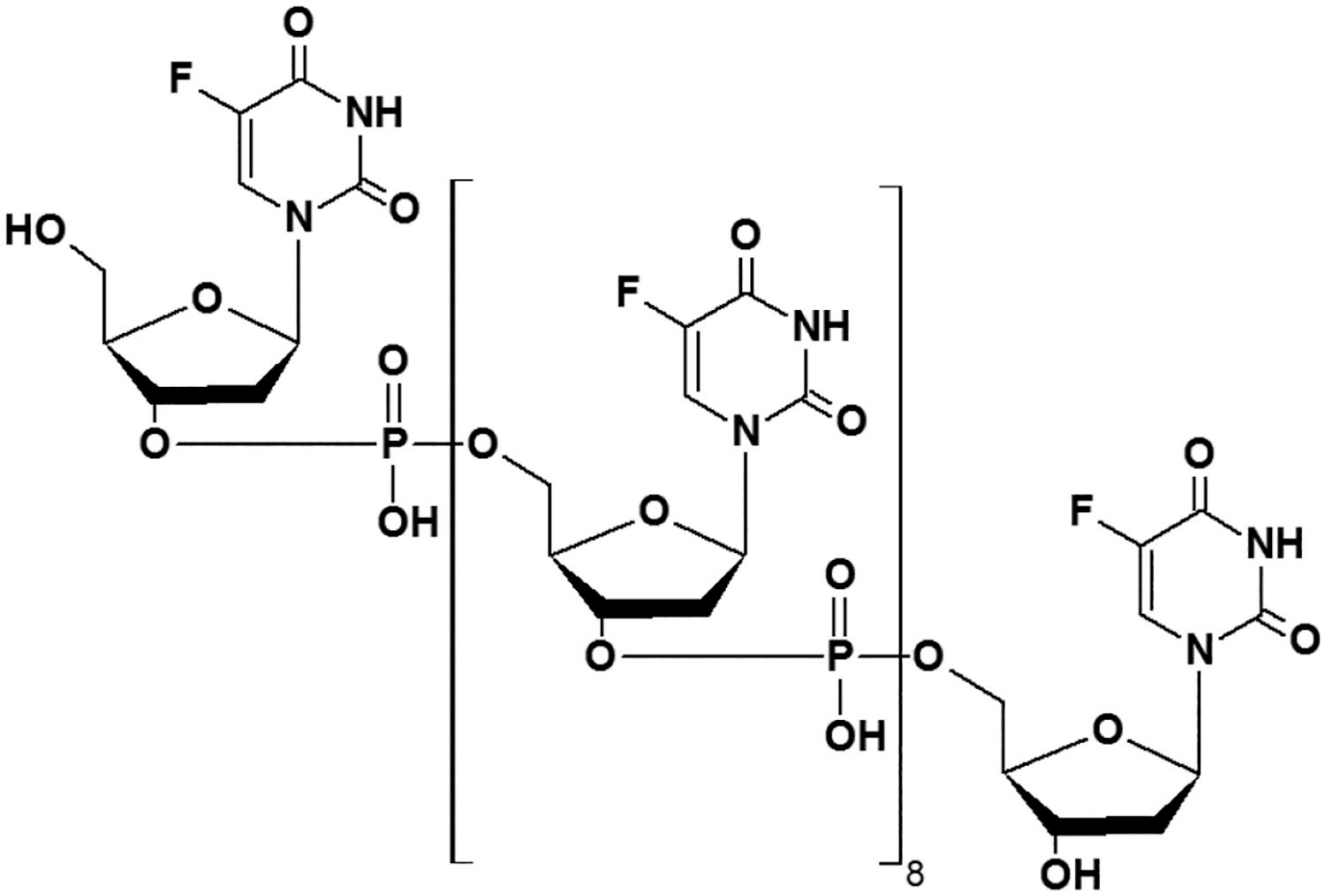
Structure of F10 a polymeric fluoropyrimidine with a DNA-directed mechanism of action

While 5-FU can affect both RNA and DNA-directed processes, the anti-cancer activity of 5-FU-based regimens correlates with thymidylate synthase (TS) expression^[22-24]^, consistent with DNA-directed effects through the 5-FU metabolite FdUMP as being central to efficacy. 5-FU is inefficiently converted to FdUMP^[25]^ which limits its inherent anti-tumor activity. To overcome limitations in efficacy due to inefficient metabolism we developed polymeric fluoropyrimidines (e.g. F10; Figure 1) with enhanced DNA-directed effects. We demonstrated F10 displays greater overall potency, improved anti-tumor activity, and reduced systemic toxicities relative to 5-FU in multiple pre-clinical models^[26]^. The potency of F10 in models of CRC that are resistant to 5-FU requires further investigation.

To gain further insight into the potential for F10 to replace 5-FU for the treatment of CRC, we have undertaken a study investigating the relative potency of F10 and 5-FU towards four isogenic human CRC cell lines derived from HCT-116 but that differ in *TP53* mutational status^[21]^. HCT-116 is a cellular model of KRAS-mutant^[27]^, MSI+ CRC^[28]^ and is considered relatively 5-FU-resistant^[29]^. HCT-116 is p53 wild-type and variants that are p53^−/−^ or that express the p53 GOF mutation R248W on one allele with the second allele either expressing WT p53 (R248W/+) or being inactivated (R248W/−) were previously developed^[21]^. The R248W mutation affects the DNA binding surface of p53^[30]^ and HCT-116 R248W/− display reduced induction of p53 relative to WT cells while induction of p21 is absent in 5-FU-treated R248W/− cells^[21]^. Our studies show that F10 is a true DNA-directed FP^[31]^ with efficient rescue only by exogenous Thy^[32]^, but not Urd. Further, F10 is considerably more potent than 5-FU to all CRC cells tested regardless of p53 mutations. The largest resistance factor (~8-fold) occurred for both 5-FU and F10 towards HCT-116 R248W/− cells indicating GOF mutations of this class confer resistance broadly to FP drugs. However, even these cells remained sensitive to F10 at sub-micromolar concentrations that are likely therapeutically achievable while the IC_50_ for 5-FU towards these cells was 69.5 ± 12.8 μmol/L, consistent with CRC tumors with this genotype being resistant to treatment with 5-FU. Our studies indicate F10 and other polymeric FPs are likely to be effective for treatment of CRC regardless of *TP53* mutational status.

## METHODS

### Cell lines and drugs

HCT-116 p53^+/+^, HCT-116 p53^−/−^, HCT-116 p53^R248W/+^ and HCT-116 p53^R248W/−^ human colon colorectal carcinoma (CRC) cell lines were supplied by GRCF Biorepository & Cell Center (Johns Hopkins University). All human CRC cell lines were maintained in Dulbecco’s Modified Eagle Medium (DMEM; Lonza, Switzerland) supplemented with 10% fetal bovine serum (FBS; Sigma, St. Louis, Missouri). Cell lines were cultured in conditions of 37 °C and 5% CO_2_. F10 (ST Pharm Co., South Korea) and 5-fluorouracil (Sigma, St. Louis, Missouri) were used for drug treatment. F10 was prepared in phosphate buffered saline (PBS) while 5-FU was prepared at 50 mg/mL in dimethyl sulfoxide (DMSO) and diluted with PBS or DMEM.

### Cell culture

Cells were seeded in 96-well plates at the appropriate density in 200 μL medium and incubated for 37 °C overnight for cell attachment. Drug concentration was determined with a UV-Vis using their molar extinction coefficient values of 0.027 μg/mL^−1^cm^−1^ for F10 and 7.07 mmol/L^−1^cm^−1^ for 5-FU. Compounds were serially-diluted in either water or DMSO (final DMSO percentage < 1%) to a stock concentration, and an equal amount of drug was added to the exponentially growing cells for each tested concentration. All drugs were filtered in 0.22 μm filter before use in cell culture.

### Cell viability and apoptosis assays

CellTiter-Glo (Promega, Wisconsin) was used to determine cell viability. Measurements were made according to the manufacturer’s instructions. Cell viability was normalized and calculated as a relative percentage of the averaged vehicle control. Caspase-Glo 8 and Caspase-Glo 3/7 were also obtained from Promega and were used according to the manufacturer’s protocol.

### Urd/Thy rescue studies

HCT116 p53^+/+^ or HCT116 p53^−/−^ cells were plated on 96-well, white, flat-bottom plates and allowed 48 h for the cells to adhere to the plate and begin to grow exponentially until reached about 25% confluency. F10 or 5-FU were added to their respective wells to give the final desired test concentrations. Exogenous uridine (Urd; Sigma, St. Louis, Missouri), thymidine (Thy; Sigma, St. Louis Missouri) or PBS was then added to their respective wells. The plates were then shaken for 2 min by hand to allow proper mixing without disrupting cell growth prior to placing them back in the incubator at 37 °C and 5% CO_2_. After 48 h in the incubator, cell viability, caspase 8, and caspase 3/7 activity was determined using respective Promega assay. The rescue experiments were done in triplicate with four data points in each experiment per tested condition. Graphs were generated using Microsoft Excel. Statistical significance was evaluated using unpaired, two-tailed *t*-tests performed using GraphPad Prism.

### Leucovorin studies

HCT116 cells were allowed to grow in folic acid free DMEM for a few months until cells reached normal doubling time of about 20 h. Ten μmol/L of leucovorin (LV; Sigma, St. Louis, Missouri) was co-treated with 5-FU and F10 for 72 h as described before along with a drug control. Cell viability was assessed using Promega’s CellTiter-Glo assay as described before. Results were plotted using GraphPad Prism.

## RESULTS

To determine if F10 [Figure 1] displays improved cytotoxicity relative to 5-FU in cellular models of CRC and to evaluate the effects of p53 mutation on drug response, F10 and 5-FU were tested in the human CRC cell line, HCT-116 and three isogenic cell lines derived from HCT-116 that differ in p53 status. In addition to HCT-116 which is p53 wild-type, we tested 5-FU and F10 in HCT-116 p53^−/−^ cells in which both alleles of the *TP53* gene were inactivated using recombinant adeno-associated virus^[21]^. Drugs were also tested in two HCT-116 variants in which one allele of *TP53* was modified to express a p53 mutation (R248W) that is associated with increased tumor aggressiveness in murine cancer models while the second allele is either maintained as wild-type or inactivated [HCT-116 p53^R248W/+^ (R248W/+) and HCT-116 p53^R248W/−^ (R248W/−)]. The R248W point mutation is in the DNA binding domain of p53^[30]^ and confers gain-of-function (GOF)^[17]^ and is one of the most frequent mutations in human CRC although its effect on drug sensitivity is unproven.

### F10 is highly potent regardless of *TP53* status

**Figure 2:**
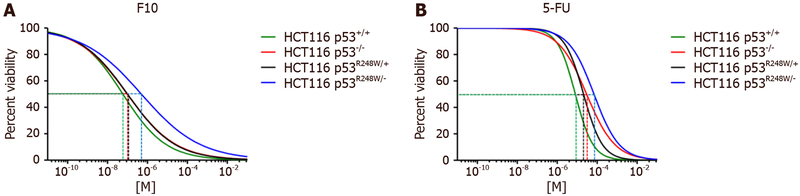
IC_50_ values. IC_50_ curve (μmol/L) after 72 h drug treatment with (A) F10 and (B) 5-FU. Experiment was done in triplicate and analyzed using GraphPad Prism (*n* = 4)

We assessed growth inhibition in the four isogenic cell lines by quantifying changes in cell viability after 72 h of drug treatment using a Cell-Titer Glo assay (Promega) which quantifies cellular ATP levels. IC_50_ values were calculated using Graphpad Prism [Figure 2] and revealed F10 was considerably more potent than 5-FU to HCT-116 cells regardless of *TP53* status [Table 1]. The improved potency of F10 relative to 5-FU ranged from 137-fold in HCT-116 cells to 304-fold in HCT-116 p53^−/−^ cells. Importantly, F10 remained highly potent to all HCT-116 cells with sub-micromolar IC_50_ values in all cases. In contrast, the IC_50_ values for 5-FU ranged from 8.07 ± 0.968 μmol/L in HCT-116 cells to 69.5 ± 12.792 μmol/L in R248W/− cells.

Resistance factors (RFs) for each of the *TP53* variant cell lines were calculated from the ratio of IC_50_ values for each p53 variant cell lines relative to parental HCT-116 cells. RF values are included in Table 1. RFs for 5-FU were significant in all cell lines and ranged from 2.63 in R248W/+ cells to 8.63 in R248W/− cells. In contrast, RFs for F10 in p53^−/−^ and R248W/+ cells were not significant, although a trend to decreased sensitivity was observed for F10 in both cell lines relative to parental HCT-116 cells. Resistance factors in R248W/− cells were significant for both F10 and 5-FU and of similar magnitude (8.42 for F10; 8.63 for 5-FU). The improved potency of F10 relative to 5-FU is preserved in HCT-116 R248W/− cells however, and these cells remain highly sensitive to F10 (IC_50_ 0.497 ± 0.206 μmol/L) but not 5-FU (IC_50_ 69.502 ± 12.792 μmol/L).

### F10 cytotoxicity is DNA-directed in p53 mutant cells

Fluoropyrimidine drugs such as 5-FU may be cytotoxic by either DNA- or RNA-mediated processes^[33]^. Our previous studies showed the cytotoxic and pro-apoptotic effects of F10 can be reversed by simultaneous co-treatment with thymidine (Thy), consistent with a DNA-directed mechanism^[32]^. In contrast, the growth inhibitory effects of 5-FU in several cell types are at least partly reversed by exogenous uridine (Urd)^[13,14]^, consistent with an RNA-mediated mechanism. Since RNA- *vs*. DNA-mediated effects may differentially affect p53 response we investigated Urd/Thy rescue in HCT-116 wild-type and p53^−/−^ cells for both F10 and 5-FU.

**Figure 3:**
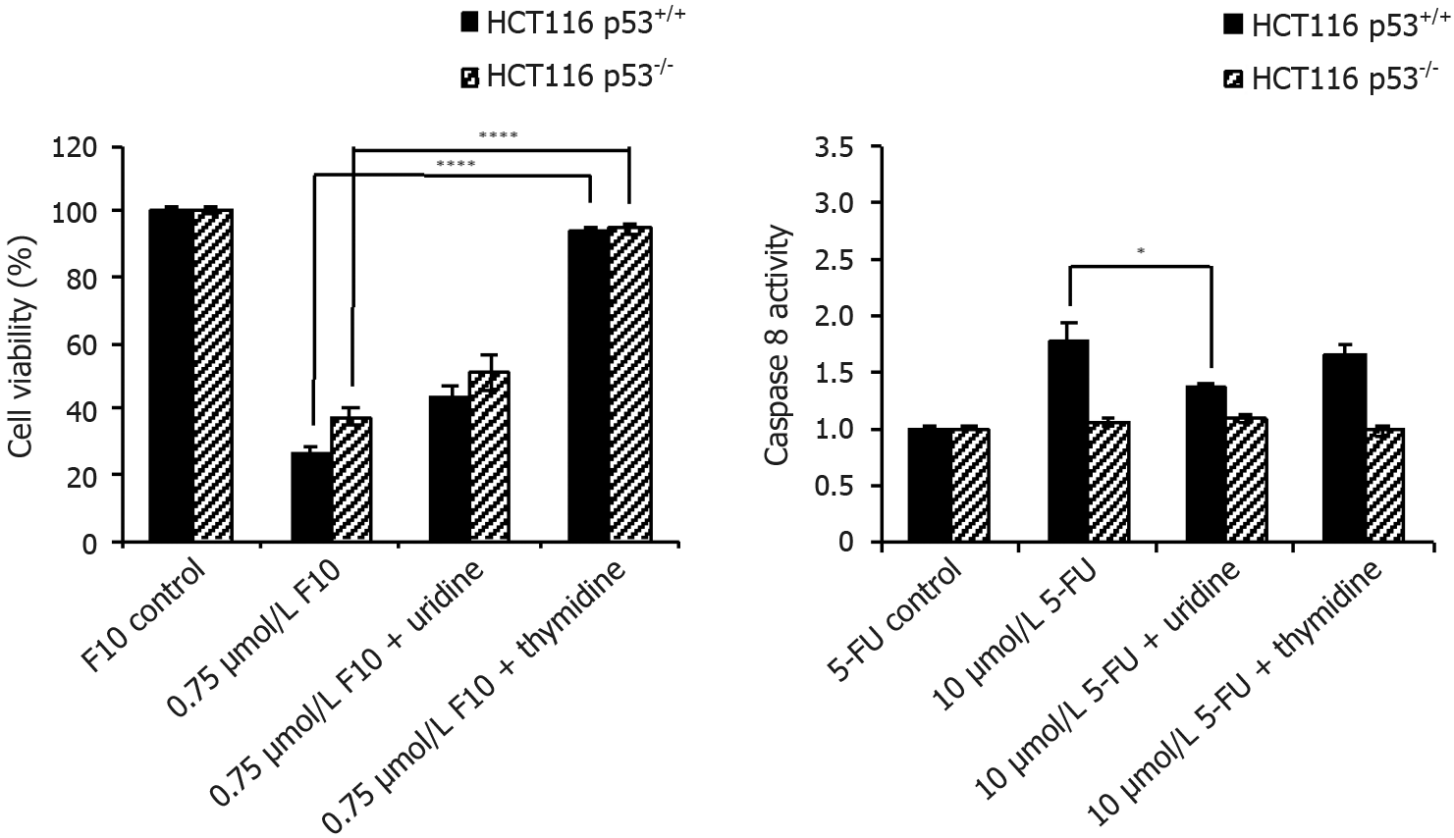
Cell viability rescue data. HCT116 p53^+/+^ and HCT116 p53^−/−^ cells were co-treated with indicated concentrations of (left) F10 or (right) 5-FU along with indicated nucleoside (uridine = 1 mmol/L, thymidine = 80 μmol/L) for 48 h. Cell viability was determined using Promega’s CellTiter-Glo ATP assay after 48 h and calculated following blank subtraction relative to respective controls. Experiments done in triplicate ± SEM. *P*-values calculated using GraphPad Prism (*n* = 4; *P ≤ 0.05, ****P ≤ 0.0001)

To determine whether the effects of F10 and 5-FU are RNA- or DNA-directed, we set up a simple rescue experiment where we provided exogenous nucleosides to cells under conditions of drug treatment and evaluated how nucleoside co-treatment affected cell viability and caspase activation. F10-treated cells were rescued from cell death when given exogenous Thy consistent with F10 being primarily DNA-directed in both wild type and p53^−/−^ HCT-116 cells and with no p53-dependence to Thy rescue [Figure 3]. Interestingly, while the rescue of F10 cytotoxicity with Urd was less than with Thy, partial rescue with Urd was observed. While the observed effect of partial Urd rescue could be due to several factors, including increased cell proliferation upon Urd co-treatment, we cannot rule out an RNA-directed component to F10 cytotoxicity that is rescued with Urd. In contrast, our data revealed no rescue of 5-FU cytotoxicity with either Urd or Thy. These results were somewhat unexpected since previous studies had demonstrated at least partial rescue of 5-FU growth inhibition by Urd^[14]^. Interestingly, while Urd had no significant rescue effect with cell viability as an endpoint, Urd did rescue the pro-apoptotic effects of 5-FU selectively in p53^+/+^ cells (see below).

**Figure 4:**
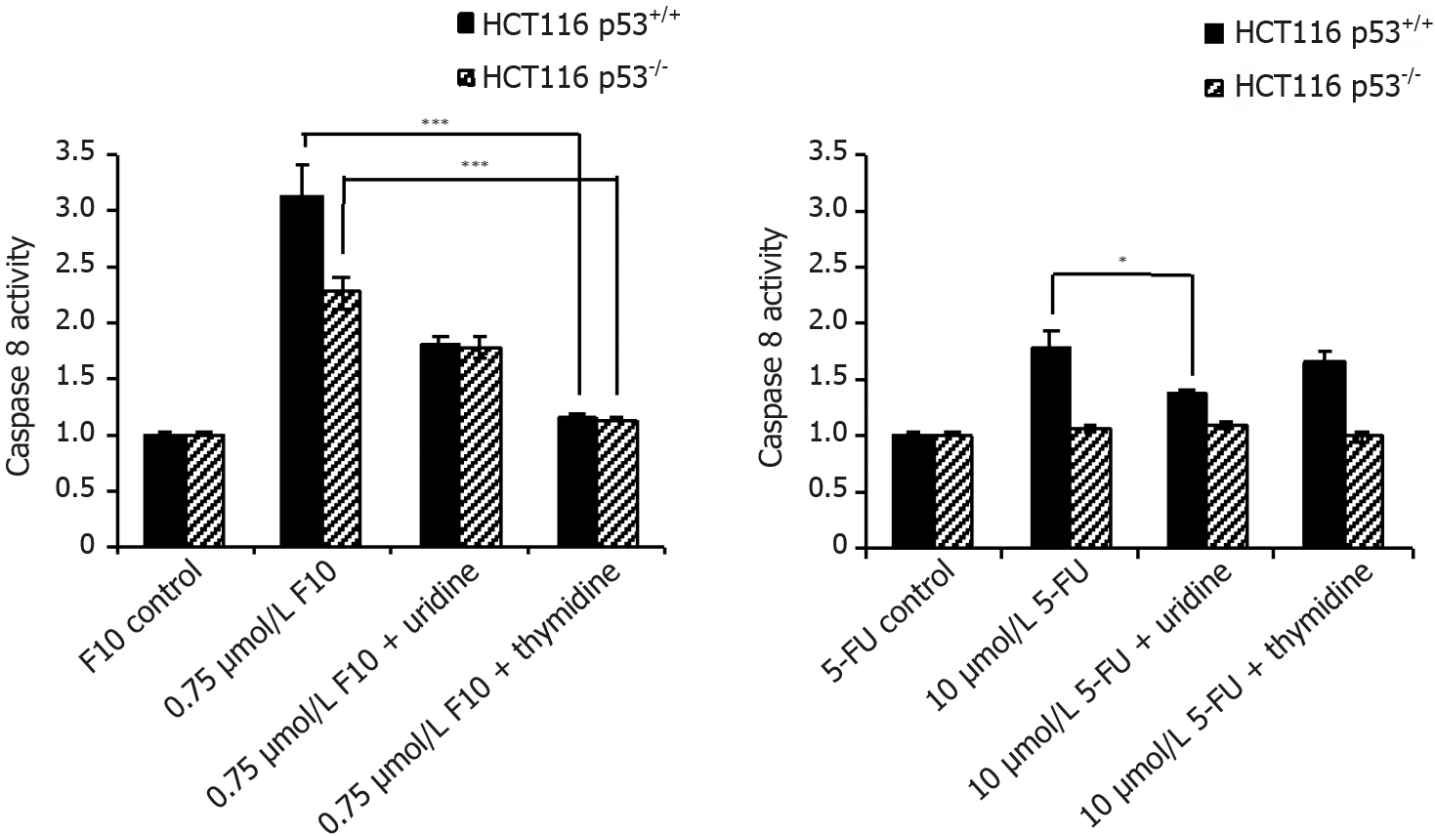
Caspase-8 activity rescue data. HCT116 p53^+/+^ and HCT116 p53^−/−^ cells were co-treated with indicated concentrations of (left) F10 or (right) 5-FU along with indicated nucleoside (uridine = 1 mmol/L, thymidine = 80 μmol/L) for 48 h. Caspase activity per cell viability was determined using Promega’s Caspase-Glo assay after 48 h and calculated following blank subtraction relative to respective control activity. Experiments done in triplicate ± SEM. *P*-values calculated using GraphPad Prism (*n* = 4; *P ≤ 0.05, ***P ≤ 0.001)

**Figure 5:**
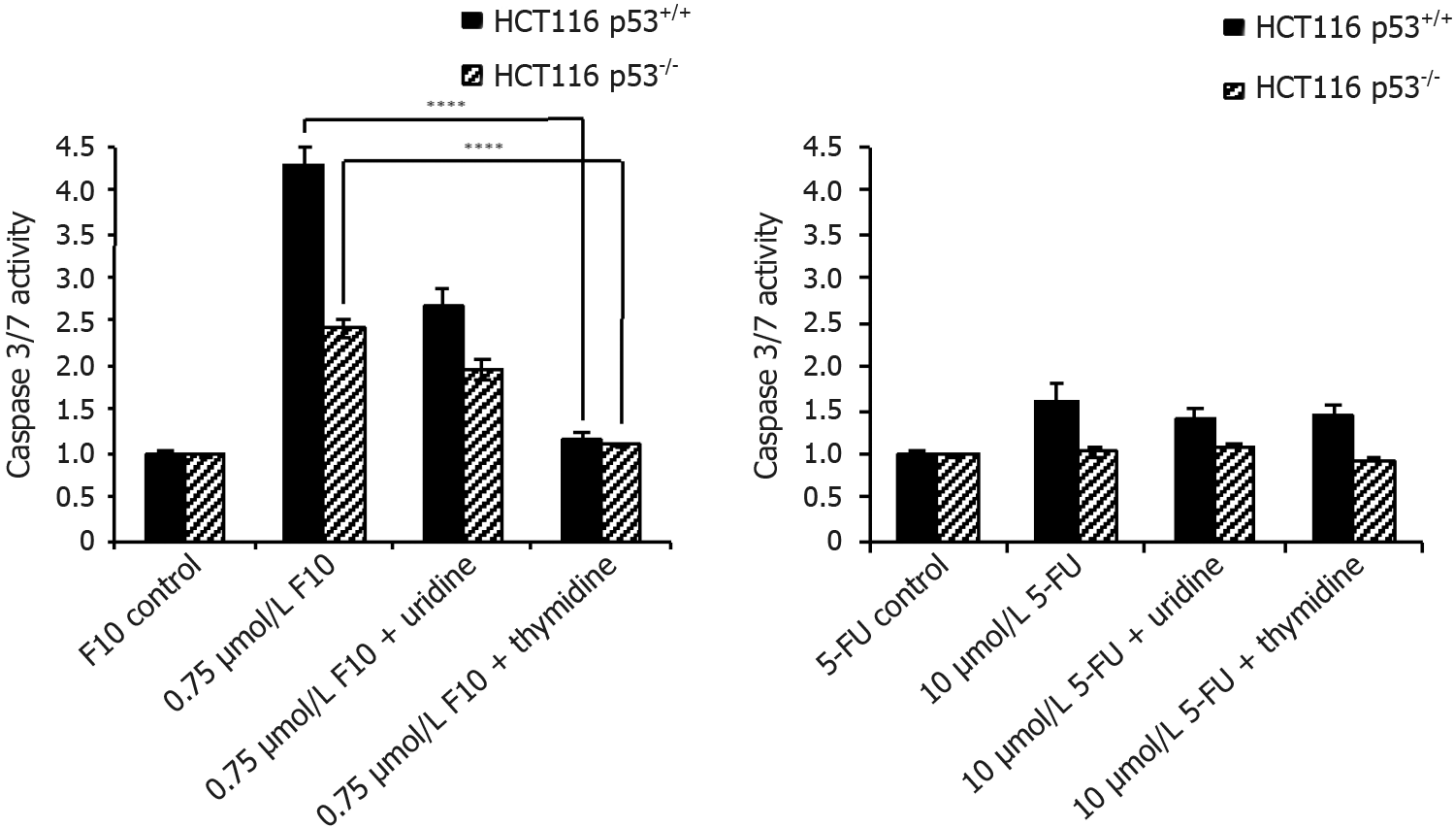
Caspase-3/7 activity rescue data. HCT116 p53^+/+^ and HCT116 p53^−/−^ cells were co-treated with indicated concentrations of (left) F10 or (right) 5-FU along with indicated nucleoside (uridine = 1 mmol/L, thymidine = 80 μmol/L) for 48 h. Caspase activity per cell viability was determined using Promega’s Caspase-Glo assay after 48 h and calculated following blank subtraction relative to respective control activity. Experiments done in triplicate ± SEM. *P*-values calculated using GraphPad Prism (*n* = 4; ****P ≤ 0.0001)

The effects of Urd/Thy rescue on F10-induced apoptosis in HCT-116 p53^+/+^ and p53^−/−^ cells were qualitatively similar to the effects of F10 on cell viability. F10 treatment resulted in increased caspase 8 [Figure 4] and caspase 3/7 [Figure 5] activities in both p53^+/+^ and p53^−/−^ cells. These results are consistent with apoptosis being induced in a p53-independent manner in HCT-116 cells in response to F10 treatment although caspase activation was somewhat greater in p53^+/+^ cells, however the extent of the decrease reflected the overall reduced sensitivity of p53^−/−^ cells to F10 and could reflect differences in cell-doubling rates or other factors unrelated to the role of p53 in mediating response to cellular damage. The pro-apoptotic effects of F10 were completely inhibited by co-treatment with Thy in both cell lines consistent with apoptosis being activated in response to the DNA-mediated effects of F10.

In contrast to the relative lack of p53-dependence for apoptosis in F10-treated cells, apoptosis in response to 5-FU treatment was only detected in p53^+/+^ cells. Further, in contrast to the lack of Urd rescue for 5-FU in cell viability assays, the pro-apoptotic effects of 5-FU were efficiently rescued by Urd, but not Thy. Further, these effects were only significant for caspase 8 while effects for caspase 3/7 were attenuated relative to caspase 8, possibly due to cells not entering end-stage apoptosis despite initial activation of caspase 8 and the extrinsic apoptotic pathway in response to 5-FU treatment.

**Figure 6:**
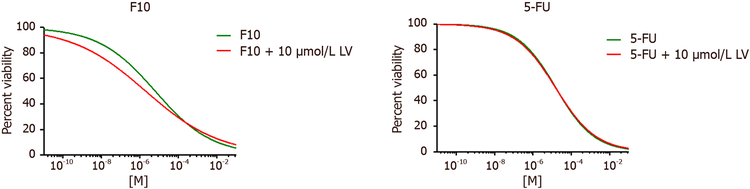
Leucovorin IC_50_ values. IC_50_ curve (μmol/L) after 72 h drug treatment with (left) F10 and (right) 5-FU co-treated with 10 μmol/L of leucovorin in folate free DMEM on wild type HCT116 cells. Data were analyzed using GraphPad Prism (*n* = 4)

The effects of 5-FU may in some cell types be accentuated to display greater DNA-directed effects by cotreatment with leucovorin (LV)^[14]^. However, in our studies co-treatment with LV had minimal effect on 5-FU cytotoxicity and in fact LV-mediated effects were larger for F10 than for 5-FU [Figure 6], although these effects were not significant for either 5-FU or F10 [Table 2]. Thus, the differences in sensitivity among the isogenic HCT-116 cells included in this study likely would not be altered if LV co-treatment were included in these studies and the results have implications for clinical management of CRC with 5-FU/LV combination therapy.

## DISCUSSION

Fluoropyrimidine drugs, predominantly 5-FU remain central to the treatment of CRC^[2]^, however drug resistance is a serious issue limiting efficacy and is a major contributing factor to the continued high mortality rate of CRC, particularly for late stage disease. While a multitude of factors are known to confer resistance to 5-FU in cell-based models including deficiency in enzymes responsible for its anabolic metabolism (e.g. OPRTase, TK)^[34,35]^, elevated TS expression^[23]^ is an established cause of clinical resistance. Thus, while 5-FU may have both RNA- and DNA-directed effects, the causal link between elevated TS and 5-FU resistance is consistent with 5-FU’s anti-tumor activity resulting primarily from DNA-directed effects and resistance resulting from insufficient production of DNA-directed metabolites. In contrast, the GI-tract toxicity of 5-FU is mainly due to RNA-directed metabolites as evidenced by their amelioration upon co-administration of Urd triacetate^[36]^. To overcome these limitations of 5-FU, we developed fluoropyrimidine polymers (e.g. F10; Figure 1) to produce predominantly DNA-directed metabolites. F10 is much more potent than 5-FU and displays improved efficacy and reduced systemic toxicities in multiple pre-clinical cancer models. The present studies confirm the improved potency of F10 relative to 5-FU in HCT-116 human CRC cells and extend these findings to include CRC cells with p53 mutations. *TP53* is frequently mutated in most types of cancer^[37]^, including CRC, and *TP53* status is an important factor in chemoresistance in a variety of cellular contexts^[38]^.

While *TP53* is frequently mutated in CRC, the significance of *TP53* gene mutations for response of CRC patients to 5-FU-based chemotherapy regimens remains under investigation. *TP53* mutation status is not currently used to direct therapy decisions^[2]^, however there is increasing evidence that not all *TP53* mutations exert equivalent effects on tumor aggressiveness or chemotherapy response. In particular, there is evidence that *TP53* mutations that confer “gain-of-function” by altering the DNA-binding domain of p53 may be particularly deleterious as they cause increased tumor aggressiveness and metastasis in animal models^[37,38]^. Our studies demonstrate that loss of p53 has only a modest effect on 5-FU cytotoxicity with a resistance factor (RF) of 3.75 for HCT-116 p53^−/−^ cells relative to p53^+/+^. The corresponding RF for F10 (1.66) was not statistically significant in these studies, a result that is consistent with the potential translation of F10 to treat CRC that is p53-null.

The RF’s for HCT-116 cells expressing the R248W GOF mutation depended heavily on whether the second allele encoded wild-type p53 or was deleted [Table 1]. HCT-116 R248W/+ cells that retain expression of one allele encoding wild-type p53 showed RFs for 5-FU similar to p53-null cells (2.63 *vs*. 3.75). For F10, the RF in R248W/+ cells was similar to p53-null cells and also not significant (1.69). In contrast, R248W/− cells in which no wild-type p53 is expressed showed larger RFs, 8.63 for 5-FU and 8.42 for F10. The extent to which these RFs confer resistance in vivo requires further investigation however there is reason to think RF’s of this magnitude pose a more significant impediment to successful treatment with 5-FU than for F10. The IC_50_ value for 5-FU in R248W/− cells is 69.5 μmol/L which in light of the serious systemic toxicities associated with 5-FU treatment^[10]^ may not be therapeutically obtainable. In contrast, F10 remains cytotoxic to these cells at sub-micromolar concentrations (IC_50_ 0.497 ± 0.206 μmol/L). Hence, the relative potency advantage of F10 relative to 5-FU is retained in R248W/− cells and F10 has strong potential to be effective towards CRC tumors with the R248W/− mutation profile even if 5-FU-based regimens are ineffective.

F10 was designed to overcome limitations associated with 5-FU due to inefficient conversion to DNAdirected metabolites and the Urd/Thy rescue experiments undertaken in the present studies confirm the relatively increased DNA-directed character for F10 relative to 5-FU. F10 cytotoxicity [Figure 3] and apoptosis [Figures 4 and 5] are both rescued upon co-treatment with Thy, a result in accord with our previous studies showing F10 cytotoxicity is DNA-directed^[31,39]^. In previous studies, we showed the rescue effects of Thy were limited to the first 16 h of treatment^[32]^ after which F10-mediated cellular damage becomes permanent and is no longer reversible with exogenous Thy. The lesser effects of Urd rescue of F10 effects observed in the present studies could be due to several factors including an effect of Urd on cell proliferation or increased Thy levels resulting indirectly from Urd treatment from residual TS activity at the F10 concentration (0.75 μmol/L) used in these studies. Somewhat unexpected in these studies was the lack of rescue of 5-FU effects with either Urd or Thy. Previous studies measured cell proliferation as an endpoint of 5-FU activity^[14]^ while our studies assessed viable cells based on ATP content. Thus the type of assay used may account for some of the observed differences. While we detected no effect of Urd rescue on 5-FU cytotoxicity [Figure 3], efficient rescue of apoptosis in 5-FU-treated cells was detected with Urd co-treatment [Figure 4] indicating the pro-apoptotic activity of 5-FU is predominantly RNA-mediated in these cells. Unlike for F10, apoptosis in response to 5-FU was entirely p53-dependent. Interestingly, the enhancement of 5-FU cytotoxicity by LV co-treatment was minimal [Figure 6], and less than the enhancement of F10 by LV. Thus, the observed differences in potency and Urd/Thy rescue for F10 relative to 5-FU are not likely affected by inclusion of LV in 5-FU-based regimens and our studies may have implications for treatment of p53mutant CRC with 5-FU/LV and related combinations. Interestingly, the increased DNA-directed effects of F10 appear to make it more susceptible than 5-FU to folate status and translational studies with F10 likely would be best pursued with LV co-treatment.

Fluoropyrimidine-based chemotherapy regimens remain central to treatment of CRC. The present studies demonstrate that *TP53* mutational status affects the inherent responsiveness of CRC cells to 5-FU. Further investigation into the clinical significance of *TP53* mutations, particularly GOF mutations in the context of loss of the wild-type allele are warranted to understand if the inherent reduction in sensitivity to 5-FU that was detected in these studies translates into reduced efficacy of 5-FU-based regimens. Importantly, our studies reveal F10 remains highly potent to CRC cells regardless of *TP53* mutational status. Thus, the use of F10 or fluoropyrimidine polymers could result in effective treatment of CRC that is resistant to 5-FU due to mutations in *TP53*.

## Figures and Tables

**Table 1:** IC_50_ values

	IC_50_ values (μmol/L ± SEM)			
	F10	F10 resistance factor	5-FU	5-FU resistance factor
HCT116 p53^+/+^	0.059 ± 0.010	0.00	8.072 ± 0.968	0.00
HCT116 p53^−/−^	0.098 ± 0.034	1.66	**29.785 ±3.919	3.75
HCT116 _P_53^R248W/+^	0.100 ± 0.033	1.69	**20.893 ± 2.469	2.63
HCT116 p53^R248W/−^	0.497 ± 0.206	8.42	**69.502 ± 12.792	8.63

IC_50_ values (μmol/L) after 72 h drug treatment of each indicated drug. Experiment done in triplicate ± SEM. Resistance factor indicates the fold increase in IC_50_ values for the indicated drug compared to the wild-type HCT116 cell line. Data analyzed using Graphpad Prism (*n* = 4;

** *P* ≤ 0.01)

**Table 2:** Leucovorin IC_50_ values

	IC_50_ values (μmol/L ± SEM)
F10	6.245 ± 1.72
F10 + 10 μmol/L LV	2.006 ± 0.732
5-FU	15.88 ± 3.16
5-FU + 10 μmol/L LV	15.53 ± 3.40

IC_50_ values (μmol/L) after 72 h drug treatment of each indicated drug and co-treated with or without 10 μmol/L LV on wildtype HCT116 cells. Experiment done in triplicate ± SEM. Data analyzed using Graphpad Prism (*n* = 4)
